# Water-repellent Hybrid Nanowire and Micro-scale Denticle Structures on Flexible Substrates of Effective Air Retention

**DOI:** 10.1038/s41598-018-35075-2

**Published:** 2018-11-09

**Authors:** Sungwon Jo, Seongbin Ahn, Heungsoo Lee, Chul-Min Jung, Simon Song, Dong Rip Kim

**Affiliations:** 1 School of Mechanical Engineering, Hanyang University, Seoul, 04763 South Korea; 2 Institute of Nano Science and Technology, Hanyang University, Seoul, 04763 South Korea; 3 The 6th R&D Institute, Agency for Defense Development, Changwon, South Korea

## Abstract

The air retention capability of a superhydrophobic surface plays the crucial role of drag reduction in an aqueous environment. Here, fabrication of water-repellent hybrid structural surfaces by synthesizing superhydrophobic nanowires with a high aspect ratio on micro-scale denticle structures to improve their air holding capacity in water is reported. The hybrid structure is realized by carrying out polymer molding of denticle structures on flexible substrates, hydrothermal growth of nanowires, and subsequent ultra-thin film coating. This technique is readily applicable to large areas, and the fabricated substrates are attachable onto curved surfaces. Our engineered, super water-repellent hybrid structures are found to effectively maintain air bubbles on their surfaces in a highly shear flow condition with a wall shear stress of up to 33.4 Pa, due to the combined effects of the micro-scale denticle structure, which reduces flow resistance, and the superhydrophobic, high-aspect-ratio nanowire structure, which enhances the capillary force to maintain the air bubbles. Our results show the importance of developing superhydrophobic structures of improved air retention capability.

## Introduction

Developing nano- and micro-scale structures on the surfaces can reduce frictional drag without additional devices or energy consumption has been actively studied for various structures, such as ships, underwater vehicles, and piping systems^1–23^. One of the ways to reduce frictional drag through surface structures is to utilize a micro-scale riblet structure which mimics the shark scale^1–12^. The micro-scale riblet structure, which is a one-directionally grooved structure along the flow direction, is known to keep energetic vortices in a high-speed turbulent flow away from the wall. Consequently, it can decrease frictional drag on the surface caused by the interaction between complex vortical motions and the surface^3^. Such drag reduction effects of the micro-scale riblet structures were validated by carrying out numerical studies^5–8^ and experimental demonstrations^9,10^. For example, micro-scale riblet structures were realized by applying laser etching method to cast acrylic, which reduced the frictional drag by 19% under channel flow conditions of a Reynolds number (*Re*) of 4,700. Coating those structures with hydrophobic nanoparticles further enhanced the frictional drag reduction up to 34% in the same flow conditions^10^.

Recently, superhydrophobic nanostructured surfaces have received considerable attention because they can induce the slippage of water at the surface where the air is trapped. This, in turn, can largely decrease the frictional drag because the viscosity of air is much smaller than that of water^13–23^. In particular, the flow over the zinc oxide nanorods coated with hydrophobic surface modifier exhibited slippage at the surface with a maximum slip length of 5 μm in a shear rate of up to 100 s^−1^ in a liquid with a viscosity of 0.01–0.06 Pa-s. The slipping phenomena disappeared above the shear rate of 100 s^−1^, corresponding to the wall shear stress of about 6 Pa for a liquid with a viscosity of 0.06 Pa-s^13^. Compared with a planar surface, superhydrophobic polycarbonate nanofur structures with a high aspect ratio improved the air holding capacity owing to a higher capillary force, thereby reducing frictional drag by ~50% on average in a channel flow with a *Re* of up to 120^14^. Superhydrophobic random hierarchical micro- and nano-scale structures were fabricated by using the anodizing method at a high temperature and subsequent hydrophobic coating. This further enhanced the air holding capability, and an excellent frictional drag reduction of up to 50% in the boundary layer flow of water in an open channel with a *Re* ranging of 1~2 × 10^5^ was observed^15^. However, such reduction effects decreased above a *Re* of 2 × 10^5^ and disappeared around a *Re* of 2.6 × 10^5^, because the air bubbles on the surface broke away. These findings show the importance of the capability of a structure to hold an air layer on its surface in a high Reynolds number flow or highly shear flow for frictional drag reduction^15^.

Considering the importance of frictional drag reduction in the highly shear flow of real applications, the engineered surface needs further development to maintain air bubbles. Here, we demonstrate the fabrication of superhydrophobic hybrid structures combining micro-scale denticle structures (*i.e*., an array of micro-scale riblet structures) with high-aspect-ratio nanowires to effectively maintain air bubbles in a highly shear flow. Specifically, we fabricate flexible polydimethylsiloxane (PDMS) micro-scale denticle structures by using a simple molding process. Then, we uniformly synthesize cobalt hydroxide carbonate nanowires on the micro-scale denticle structures by using hydrothermal growth, followed by thin-film hydrophobic coating. Our method is scalable and repeatable, and the fabricated super water-repellent substrates are flexible and attachable onto curved surfaces. Importantly, the air retention capability of the super water-repellent hybrid structures is discussed with air bubble visualization results obtained under highly shear flow conditions. Our results show the promise of developing superhydrophobic hybrid surface structures with effective air retention.

## Results

### Fabrication of water-repellent hybrid nanowire and micro-scale denticle structures

**Figure 1:**
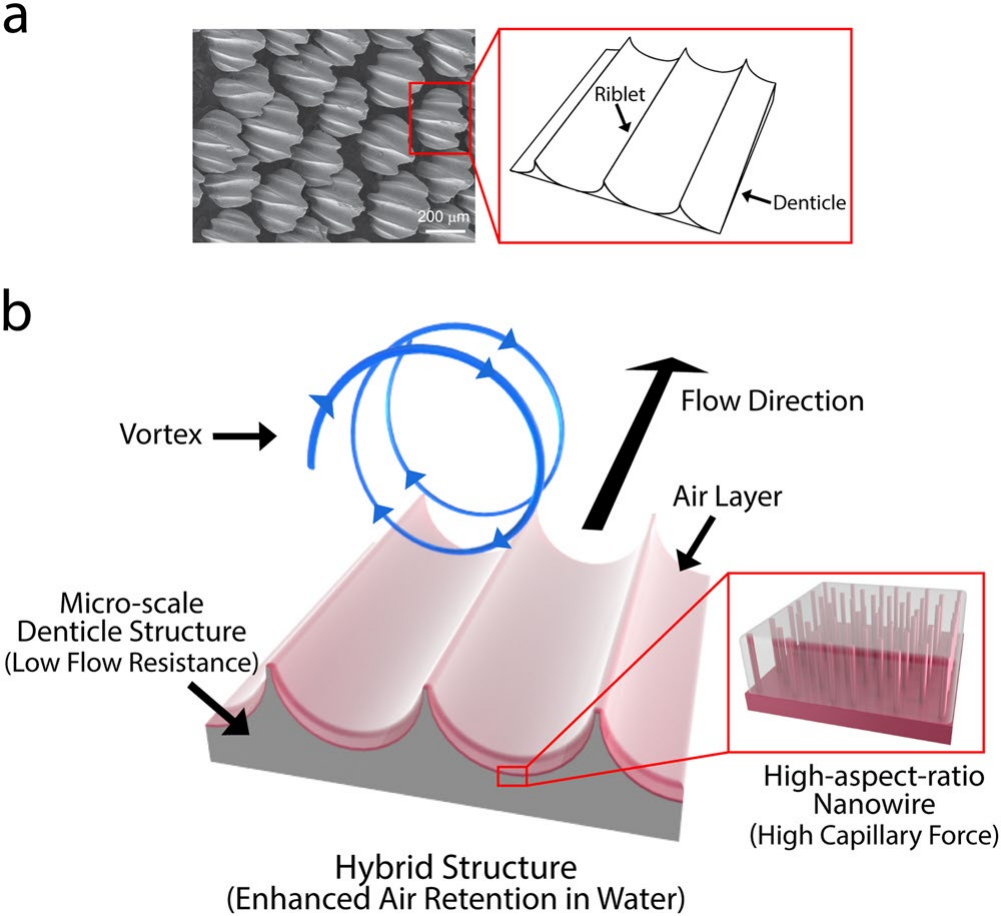
Micro-scale structures of shark skin and the schematics of our water-repellent hybrid nanowire and micro-scale denticle structures. (**a**) Scanning electron microscopy (SEM) top view image (left) of shark skin replica and graphical image (right) of micro-scale dermal denticle and riblet structures. The SEM image of shark skin is adapted from Manoonpong *et al*.^24^. The micro-scale denticle structure contains micro-riblet structures along the flow direction. (**b**) Schematics of air layer holding enhancement of our water-repellent hybrid structure. While the high-aspect-ratio nanowires hold the air layer very well, the micro-scale denticle structure keeps the energetic vortices away from the surface, which enhances the air holding capacity of the hybrid structures.

**Figure 2:**
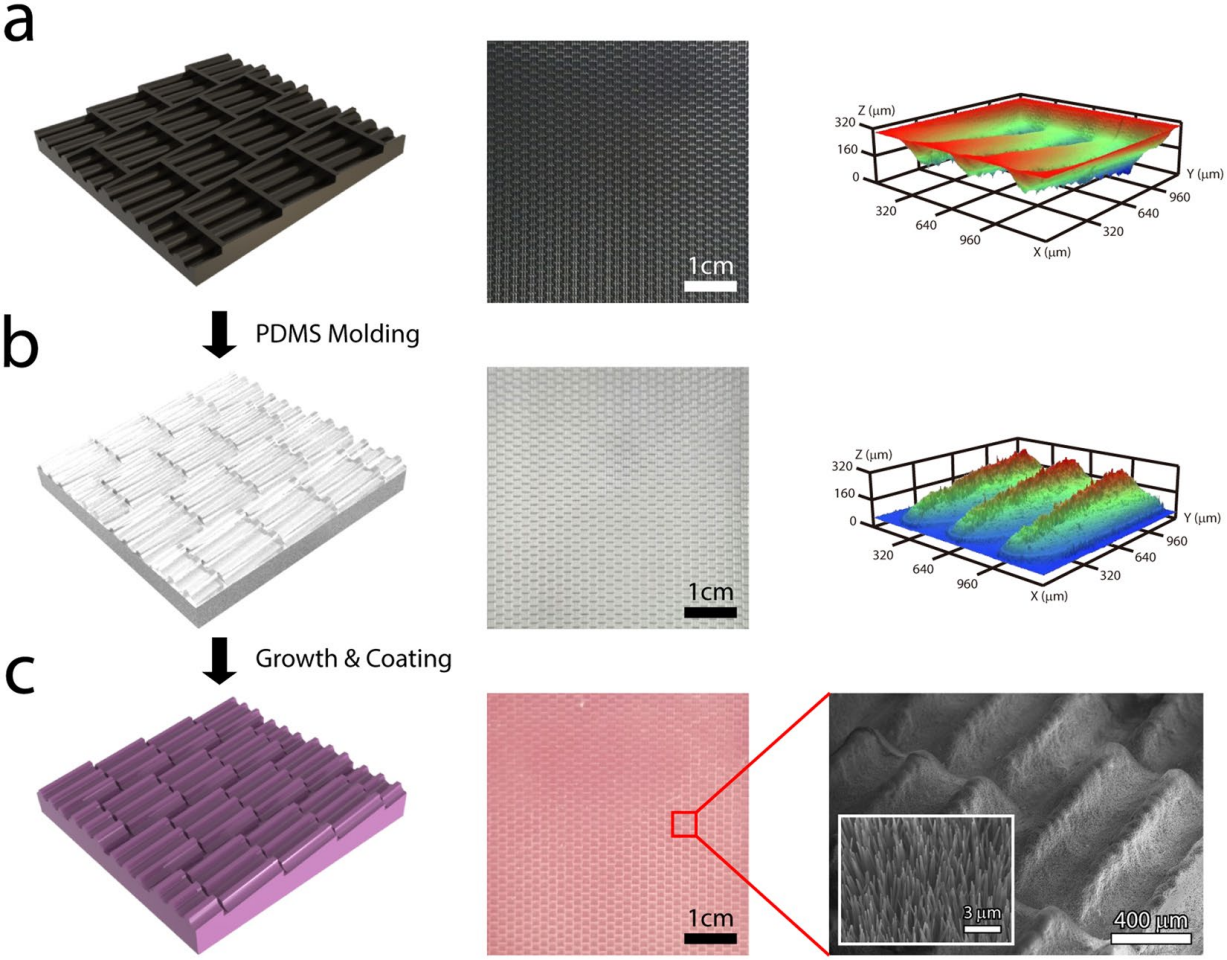
Fabrication procedure and surface morphology of water-repellent hybrid nanowire and micro-scale denticle structures. We first fabricate the mold by using laser processing, and then carry out PDMS molding to generate micro-scale denticle structures on flexible substrate. Finally, we synthesize high-aspect-ratio nanowires on the molded substrate, followed by PTFE ultra-thin film coating. Schematic (left), photograph (middle), and three-dimensional surface morphology (right) of (**a**) the metallic mold to generate the micro-scale denticle structures are fabricated by laser processing and of (**b**) the fabricated flexible polydimethylsiloxane (PDMS) substrate with micro-scale denticle structures by molding. (**c**) Schematic (left), photograph (middle), and SEM images (right) of water-repellent hybrid structures on flexible substrates synthesized by hydrothermal synthesis and consequent PTFE ultra-thin film coating. The high-magnification SEM image (inset) shows the high-aspect-ratio nanowire structures formed on the micro-scale denticle structures.

To enhance the air holding capacity of a surface in a highly shear flow, we combine flow-resistance-reducing micro-scale structures with effective-air-holding nano-scale structures. We mimic the shark skin which consists of micro-scale dermal denticle including several riblet structures aligned along the flow direction (Fig. 1a)^24^. Then, we position superhydrophobic, high-aspect-ratio nanowires on the micro-scale denticle structures, which effectively grab the air layer in a highly shear flow (Fig. 1b). Figure 2 illustrates the fabrication process and surface morphology of our water-repellent hybrid nanowire and micro-scale denticle structures. First, to mimic shark skin, we fabricate the metallic mold (steel, NAK80) by using laser processing to make the micro-scale denticle structures (a width of 1.2 mm and a length of 1.2 mm) in which three riblet structures (a width of 100 μm, a height of 150 μm, and a period of 400 μm) are arranged (Fig. 2a). It should be noted that while the shapes and dimensions of the denticle structures of shark skin are different with respect to the type of sharks, three or five riblet structures are normally formed on a denticle structure. We simplify the shape of a denticle structure as a square to contain three riblet structures. The dimensions of the riblet structures are based on previous studies on effective flow resistance reduction, considering the resolution of the laser processing and the molding processing^4,12^. The micro-scale denticle structures are arranged with gaps (100 μm), and each denticle structure is inclined at seven degrees. Second, we carry out PDMS molding over the mold to generate a flexible substrate with micro-scale denticle structures. The three-dimensional surface morphologies obtained by confocal laser scanning microscopy (CLSM, Olympus, OLS4100) reveal that the micro-scale denticle structures are well-realized, following the mold design (Fig. 2b). Third, cobalt hydroxide carbonate nanowires (a length of 10–15 μm and a diameter of 90–130 nm) are synthesized over the flexible PDMS substrate with micro-scale denticle structures by using hydrothermal growth. Detailed fabrication procedures are described in Methods Section. After the hydrothermal growth, the samples are thoroughly rinsed with deionized water and dried by blowing air. Finally, the nanowires on flexible PDMS substrates with micro-scale denticle structures are coated with a polytetrafluoroethylene (PTFE) ultra-thin film. Scanning electron microscopy (SEM, Hitachi, S-4800) images indicate that the shapes of the nanowires are maintained after the ultra-thin coating (Fig. 2c). It should be noted that the shapes of micro-scale denticle structures are also well-maintained after the synthesis of the nanowires and the film coating (Fig. S1, Supplementary Information).

### Water- and oil- repellent properties and flexibility of hybrid structures

**Figure 3:**
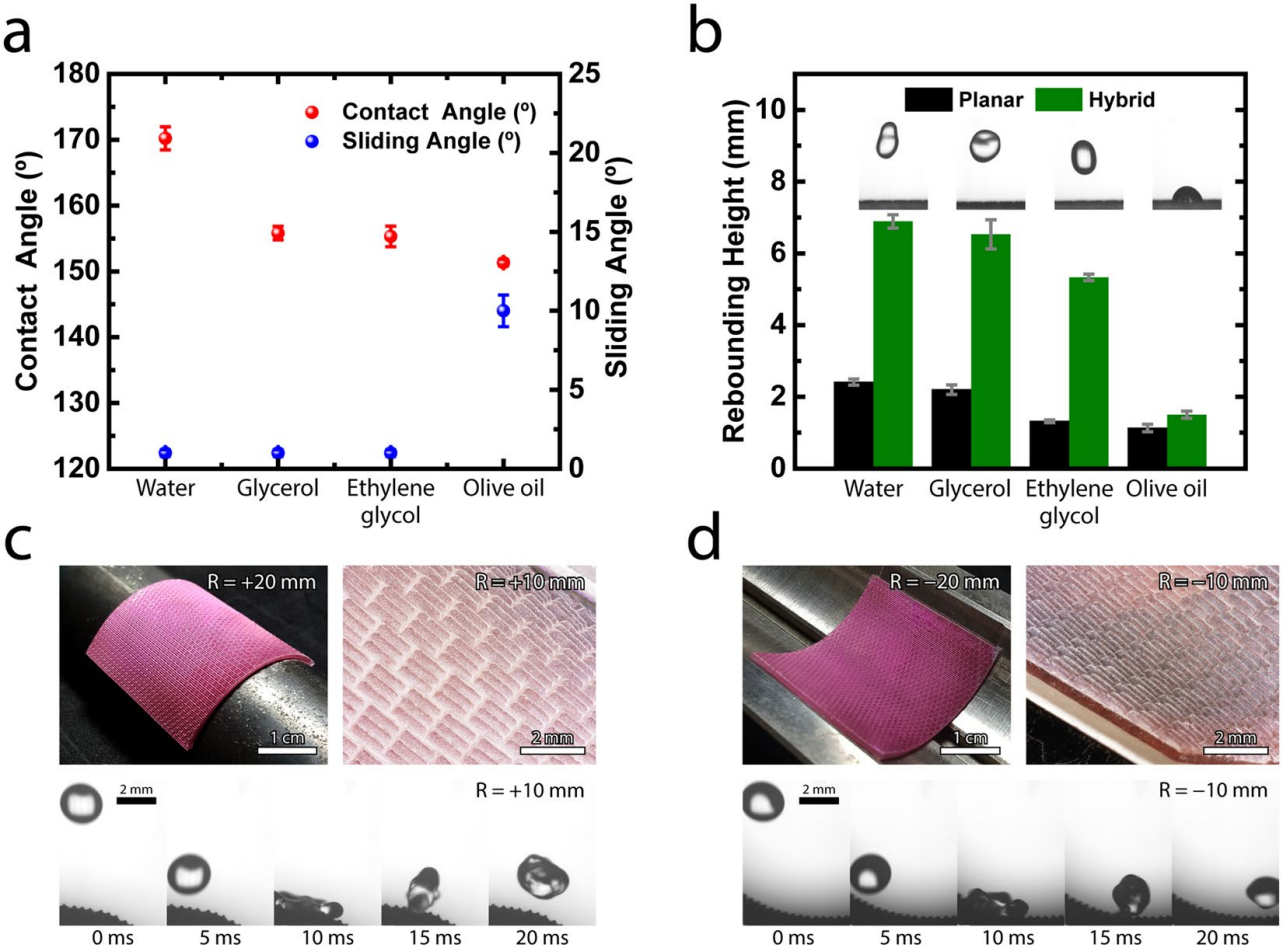
Water- and oil-repellent properties of hybrid structures. (**a**) Static contact angles (red) and sliding angles (blue) of various liquids, such as water, glycerol, ethylene glycol, and olive oil, on the hybrid structures. (**b**) Dynamic droplet behaviors of the hybrid structures. The rebounding height of a 10 μl droplet from a height of 30 mm is measured by using a high-speed camera at 2,000 frames per second. The test is repeated five times for each water, glycerol, ethylene glycol and olive oil. The images inside the graph are captured when each droplet reached its peak. The rebounding heights of water and glycerol are not significantly different (*P* = 0.17, two-tailed t-test), while others are significantly different (*P* < 0.05, two-tailed t-test). (**c**,**d**) Photographs (top, left), optical microscopy images (top, right), and dynamic droplet behaviors (bottom) of the hybrid structures attached onto (**c**) the convex surfaces and (**d**) the concave surfaces with the curvature radius of R. Water droplets are dropped from a height of 30 mm.

Our hybrid structures clearly exhibit water- and oil-repellent properties (Fig. 3). We first measure the static contact angles and sliding angles of the substrate with hybrid structures by placing 8 μl of droplets of various liquids onto the surface (Surface Electro Optics, Phoenix-10): water (surface tension of 72.1 mN m^−1^), glycerol (surface tension of 64.0 mN m^−1^), ethylene glycol (surface tension of 47.3 mN m^−1^), and olive oil (surface tension of 32.0 mN m^−1^)^25^. The contact and sliding angles are 170.2 ± 1.8° and less than 1° for water droplets and are 151.3 ± 0.5° and less than 10° for olive oil droplets. These indicate the superomniphobic properties of our hybrid structures (Figs 3a and S2, Supplementary Information). The super water- and oil-repellent properties are attributed to the morphology of high-aspect-ratio nanowires and the low surface energy of the ultra-thin coating layer. It should be noted that oil-repellent structures are realized by additional modification of the self-assembled monolayer (SAM) on the hybrid structure (see Methods Section). In addition, we monitor the dynamic droplet behavior to see the water-repellent and oil-repellent properties because the hybrid structures are utilized in a dynamic flow condition (Figs 3b and S3, Supplementary Information). Specifically, we measure the rebounding height of a 10 μl droplet from a height of 30 mm by using a high-speed camera at 2,000 frames per second. The rebounding height of water, glycerol, ethylene glycol and olive oil droplets on our hybrid structures are 6.9 ± 0.2 mm, 6.5 ± 0.4 mm, 5.3 ± 0.1 mm and 1.5 ± 0.1 mm, respectively. The water, glycerol, and ethylene glycol droplets are completely rebounded by the air layer in the hybrid structures on the substrate, although the rebounding heights of glycerol and ethylene glycol droplets are slightly lower than the rebounding height of water. However, the hybrid structure cannot completely rebound the oil droplet, which implies that the closed-cell type micro-structures are needed to rebound the oil droplets^26^. Importantly, we carried out the salt water immersion test by soaking our hybrid sample in 5 wt% sodium chloride solution for a month and have confirmed the water contact angle of 168.8 ± 1.6° and surface energy of 0.02–0.50 mJ m^−2^ ^27,28^, meaning that the wettabilities of the hybrid structures are maintained (Fig. S4, Supplementary Information).

Figure 3c,d show that the water-repellent flexible substrates can be attached to curved surfaces without sacrificing their water repellency. Optical microscopy images (Olympus, DSX 110) show that our water-repellent hybrid structures are conformally attached on the convex and concave surfaces (a curvature radius of 10 ~ 20 mm) without significant deformation of the micro-scale riblet structures in the denticle structures. It should be noted that considering the optimum geometries of the micro-scale riblet structures for flow resistance reduction, maintaining the riblet structural dimensions is important^4^. In addition, maintaining the nanowire structures without the fall-off under the deformation can be attributed to the flexible micro-scale structures on the substrate^29^. To confirm the superhydrophobicity after attaching our substrate onto the curved surfaces, we investigate dynamic droplet behavior on the curved surface (Figs 3c,d and S5, Supplementary Information). Experimental results show that water droplets are rebounded without wetting, implying that the air layer and corresponding superhydrophobic property remain similarly in dynamic conditions even after the application to convex and concave surfaces. The flexible and attachable properties of our water-repellent hybrid structures show that they can be applied to various structures, including pipes for transporting a fluid, curved surfaces of a ship and an underwater vehicle.

### Air holding capacity of hybrid structures in dynamic flow conditions of water

**Figure 4:**
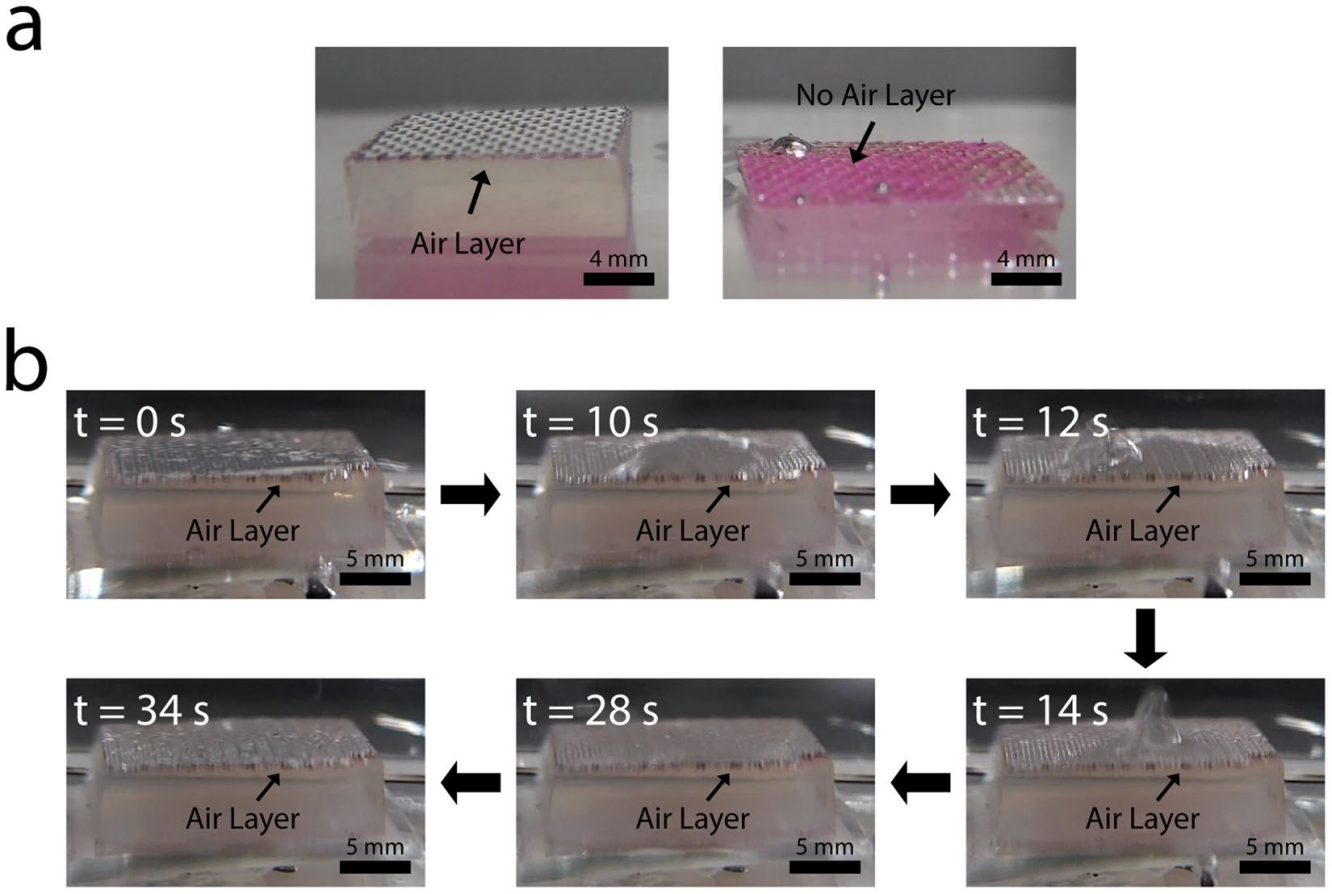
Air layer holding of hybrid structures in dynamic flow conditions of water. (**a**) Photographs of the substrate with air layer holding (left) and without air layer (right) immersed in water. The images clearly show the difference between two cases. (**b**) Air holding capacity of hybrid structures in dynamic flow conditions of water. The hybrid structure effectively grabs the air layer in water under flow disturbances. The flow is disturbed by rotating the screw at 600 rpm above the structure in the water.

The air holding capacity of superhydrophobic surfaces is crucial to frictional drag reduction. To see the air holding capacity of our hybrid structures, we first carry out the simple experiments to monitor the air layer held on the substrate with the hybrid structures in dynamic flow conditions of water (Fig. 4). Specifically, we first immerse our sample (area of 2 cm × 2 cm) in the water of 250 ml beaker. The sample is attached on the bottom of the beaker. Then, we swirl the water in the beaker by rotating a screw at 600 rpm for 30 seconds from the top side to monitor the change of air layer. The distance between the screw and the sample is about 4 mm. The air retention of the sample is clearly identified, compared to the control sample with no air layer (Fig. 4a). In a dynamic condition, the macroscale air bubbles on our sample is well-maintained under the applied disturbances (Fig. 4b; Videos S1 and S2, Supplementary Information), suggesting high air holding capacity of the hybrid structures in water.

### Air holding capacity in the controlled dynamic flow conditions of water

**Figure 5:**
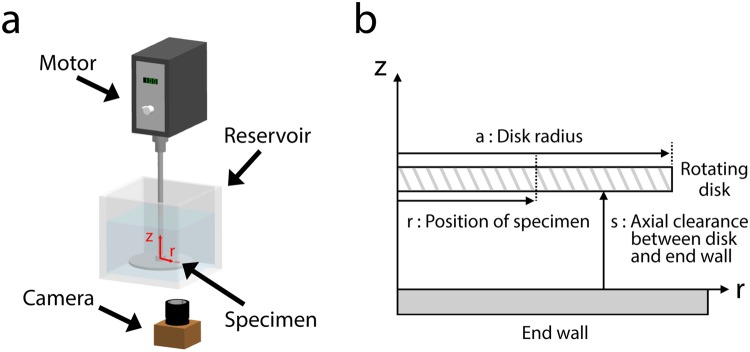
Schematics of the experiments to quantify the air holding capacity of substrates in the controlled dynamic flow conditions. (**a**) Schematic of measurement apparatus. A disk on which the upside-down sample is attached is immersed in water with a clearance of 3 mm from the bottom of the container. The disk is rotated by a motor at constant speeds for 5 min. After the rotation is stopped, the air bubbles on the substrate are monitored by using a high-resolution camera. (**b**) Schematic of geometric parameters to determine disk local Reynolds number (*Re*_*r*_) and wall shear stress (*τ*_*w*_) in dynamic flow conditions.

To quantify the air holding capacity of the hybrid structure in the controlled dynamic flow conditions, we investigate the air holding capacity of various surface structures with a rotating disk apparatus (Fig. 5)^30–32^. A disk with a radius of 60 mm is immersed in water with an axial clearance (3 mm) from the container bottom (Fig. 5a). The disk is rotated with a motor at constant speeds for 5 min, and then rotation is stopped. The rotating speeds are controlled with an increment of 100 rpm. While considering the geometries of the experimental apparatus (Fig. 5b), the corresponding disk local Reynolds number (*Re*_*r*_) and wall shear stress (*τ*_*w*_) values are defined as follows^33,34^.

1 $${\boldsymbol{R}}{{\boldsymbol{e}}}_{{\boldsymbol{r}}}=\frac{{\boldsymbol{\omega }}{{\boldsymbol{r}}}^{2}}{{\boldsymbol{\nu }}}$$

2 $${{\boldsymbol{C}}}_{{\boldsymbol{f}}}=\frac{{\bf{0.642}}}{{{\boldsymbol{r}}}^{0.1}{\boldsymbol{R}}{{\boldsymbol{e}}}_{{\boldsymbol{r}}}^{0.5}}\,{\rm{for}}\,{\rm{a}}\,{\rm{laminar}}\,{\rm{flow}}$$

3 $${{\boldsymbol{C}}}_{{\boldsymbol{f}}}=\frac{{\bf{0.0204}}}{{{\boldsymbol{r}}}^{0.1}{\boldsymbol{R}}{{\boldsymbol{e}}}_{{\boldsymbol{r}}}^{0.2}}\,{\rm{f}}{\rm{o}}{\rm{r}}\,{\rm{a}}\,{\rm{t}}{\rm{u}}{\rm{r}}{\rm{b}}{\rm{u}}{\rm{l}}{\rm{e}}{\rm{n}}{\rm{t}}\,{\rm{f}}{\rm{l}}{\rm{o}}{\rm{w}}$$

4 $${{\boldsymbol{\tau }}}_{{\boldsymbol{w}}}=\frac{1}{2}{\boldsymbol{\rho }}{(r{\boldsymbol{\omega }})}^{2}\times {{\boldsymbol{C}}}_{{\boldsymbol{f}}}$$

where *Re*_*r*_ is disk local Reynolds number, *ω* is angular velocity [s^−1^], *r* is position of specimen [m], *v* is kinematic viscosity of water [m^2^ s^−1^], *C*_*f*_ is friction coefficient, *τ*_*w*_ is wall shear stress [Pa], and *ρ* is density of water [kg m^−3^]. A specimen has an area of 11.6 × 11.6 mm^2^, and *r* is 35 mm. A high-resolution camera captures the images of the superhydrophobic surfaces before and after disk rotation to evaluate the air holding capacity.

**Figure 6:**
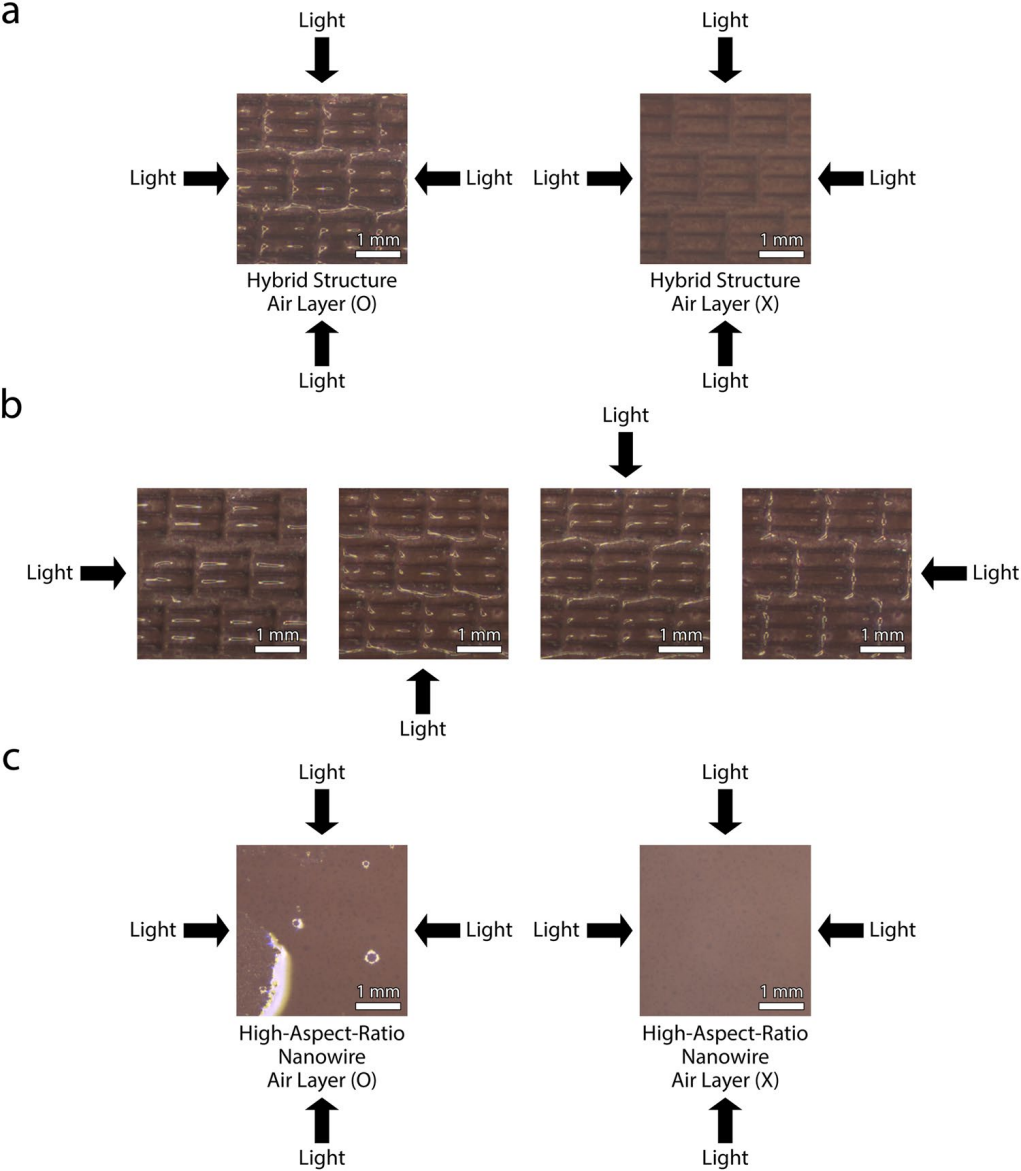
Photographs confirming the existence of air layer on hybrid structures and superhydrophobic, high-aspect-ratio nanowires in water. (**a**) Hybrid structures with air layer holding (left) and without air layer holding (right) while lighting in all directions. Both images are clearly different. (**b**) Photographs of air holding by hybrid structures in water with different lighting. The shiny parts of the images move according to the lighting directions. (**c**) Superhydrophobic, high-aspect-ratio nanowires with air layer holding (left) and without air layer holding (right) while lighting in all directions.

We first verify the presence of an air layer by comparing captured images of surfaces immersed in water (Fig. 6). The image of hybrid structures with air layer has shiny parts while hybrid structures with no air layer by degassing do not have those (Fig. 6a). These shiny parts indicate the existence of air layer because these are the result of light reflection from the interface between the air layer and water. Furthermore, we confirm the air layer holding of hybrid structures by investigating the photographs with respect to lighting variation (Fig. 6b). Depending on the lighting, the shiny parts of the images are changed, which indicate that the air layer exists on each micro-scale denticle structures. Clear differences are also shown in the experiment with superhydrophobic, high-aspect-ratio nanowires with and without air layer holding (Fig. 6c).

**Figure 7:**
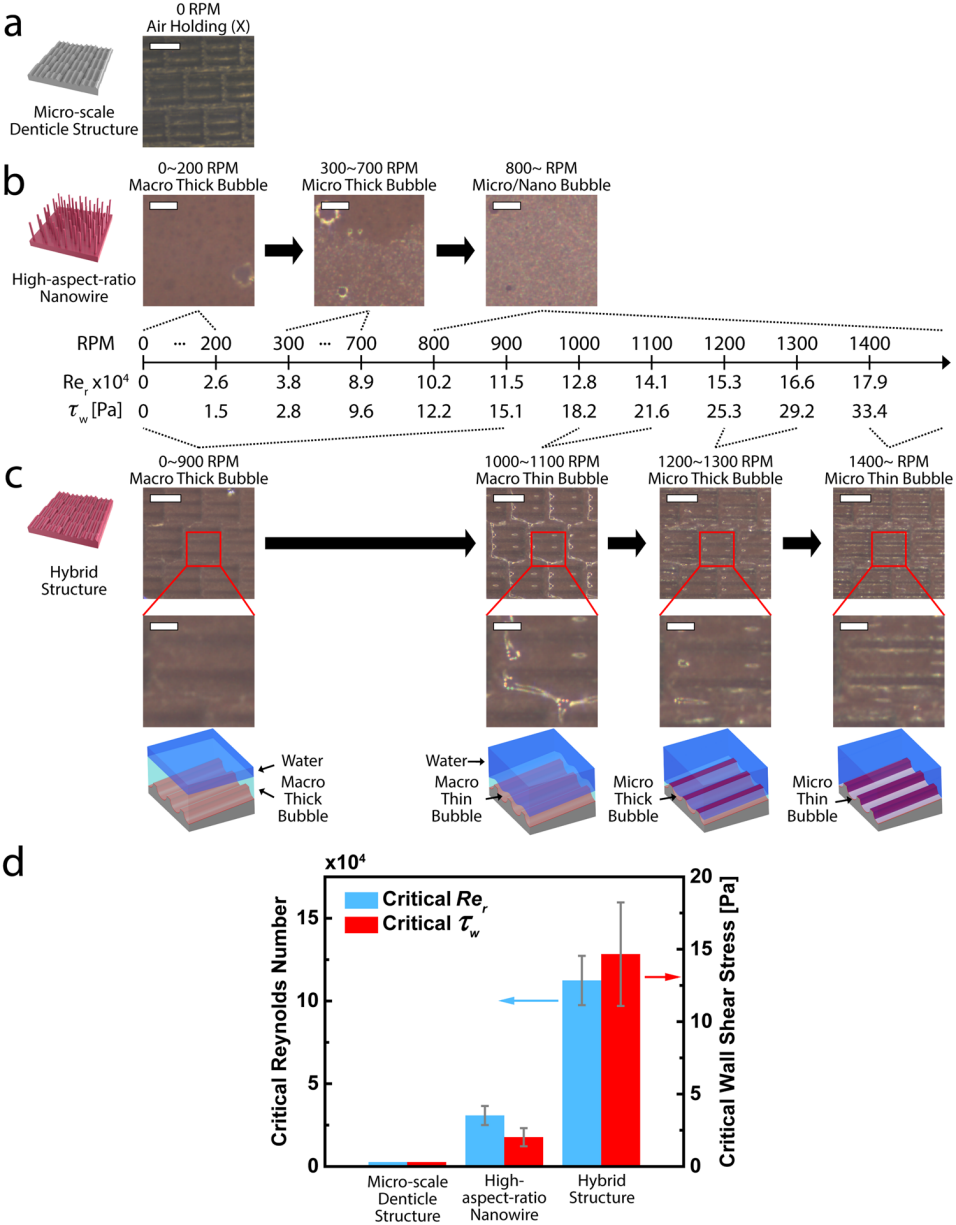
Air holding capacities of various surface structures in dynamic flow conditions of water. (**a**) Hydrophobic micro-scale denticle structures cannot retain the air layer in the water even with small disturbances (scale bar = 1 mm). (**b**) Superhydrophobic, high-aspect-ratio nanowires maintain macroscale thick bubbles on their surfaces under the flow conditions of *τ*_*w*_ up to 1.5 Pa (*Re*_*r*_ of 2.6 × 10^4^) and hold microscale thick bubbles under the flow conditions of *τ*_*w*_ up to 9.6 Pa (*Re*_*r*_ of 8.9 × 10^4^) (scale bar = 300 μm). (**c**) Hybrid structures can sustain the macroscale air bubbles on their surfaces under the flow conditions of *τ*_*w*_ up to 21.6 Pa (*Re*_*r*_ of 14.1 × 10^4^), while microscale thick bubbles can be maintained on the hybrid structures under the flow conditions of *τ*_*w*_ up to 29.2 Pa (*Re*_*r*_ of 16.6 × 10^4^). (scale bar = 1 mm, scale bar of high magnification images = 300 μm). (**d**) Critical Reynolds number (blue) and critical wall shear stress (red) of each structure which are defined as the maximum *Re*_*r*_ and *τ*_*w*_ of flow condition where macroscale air bubbles are maintained, respectively. *P* < 0.05, two-tailed t-test.

We compare the air holding capabilities of three different types of substrates: hydrophobic micro-scale denticle structures, superhydrophobic high-aspect-ratio nanowires, and superhydrophobic hybrid structures (Fig. 7). We first describe the representative air holding capabilities of each type of a substrate as follows. The micro-scale denticle structures with water contact angle of 140.0 ± 0.3° and surface energy of 3.22–3.25 mJ m^−2^ (Table S1, Supplementary Information)^27,28^ cannot retain the air layer in the water even with small disturbances, although those surfaces are treated as hydrophobic by using PTFE thin-film coating (Fig. 7a). On the other hand, superhydrophobic, high-aspect-ratio nanowires with water contact angle of 152.2 ± 0.3° and surface energy of 0.79–0.85 mJ m^−2^ (Table S1, Supplementary Information)^27,28^ shown in Figs 7b and S6 (Supplementary Information) maintain macroscale air bubbles covering the entire surface under the flow conditions up to *τ*_*w*_ of 1.5 Pa (*Re*_*r*_ of 2.6 × 10^4^). Due to the focus of the camera on the air layer, the structures are not clearly shown, which evidences the existence of macroscale air bubbles. However, in higher dynamic flow conditions of *τ*_*w*_ of 2.8 Pa (*Re*_*r*_ of 3.8 × 10^4^), the macroscale air bubbles on the nanowires are changed to the microscale thick air bubbles as such some of the surface structures become to be clearly shown, and after which, the microscale thick air bubbles on the nanowires disappear around *τ*_*w*_ of 12.2 Pa (*Re*_*r*_ of 10.2 × 10^4^) (Figs 7b and S6, Supplementary Information). We further test the air holding capacity of the hybrid structures as shown in Figs 7c and S7 (Supplementary Information). Remarkably, the macroscale air bubbles on the hybrid structures are well maintained under the flow conditions up to *τ*_*w*_ of 21.6 Pa (*Re*_*r*_ of 14.1 × 10^4^). The enhanced air holding capacity of the hybrid structures is attributed to the micro-scale denticle structures which can keep the energetic vortices away from their surfaces, reducing the external force applied to the air layer. Interestingly, as we increase *τ*_*w*_ and *Re*_*r*_ in flow conditions, the air holding behavior of the hybrid structures is transitioned from macroscale thick and thin bubbles to microscale thick and thin bubbles. The microscale thick bubbles indicate that only ridge parts of the micro-scale riblet structures become to be clearly shown, meaning that the ridge are in contact with water, whereas the microscale thin bubbles illustrate that most slope parts of the micro-scale riblet structures are clearly shown except for the valley. In both cases, the air bubbles still exist.

We define the maximum *τ*_*w*_ and *Re*_*r*_ of a flow in which macroscale air bubbles are maintained as a critical *τ*_*w*_ and *Re*_*r*_, respectively, and test five samples for each type of substrate. As a result, the critical *τ*_*w*_ and *Re*_*r*_ of our hybrid structures are in the range of 12.2~21.6 Pa (14.7 ± 3.6 Pa) and 10.2~14.1 × 10^4^ (11.2 ± 1.5 × 10^4^), respectively, while those of nanowires are in the range of 1.5~2.8 Pa (2.0 ± 0.6 Pa) and 2.6~3.8 × 10^4^ (3.1 ± 0.6 × 10^4^), respectively (Fig. 7d). Therefore, the hybrid structures can maintain the air layers about seven and four times better than the high-aspect-ratio nanowires in terms of *τ*_*w*_ and *Re*_*r*_, respectively. The superior air holding capacity of the hybrid structures is attributed to the combined effects of the micro-scale denticle structures to effectively minimize highly energetic vortices to directly contact the surface and of the high-aspect-ratio nanowires to improve the holding of air bubbles at various scales. In general, the wall shear stress of Re of 1.0 × 10^6^ for the turbulent boundary layer flow of flat plate with a characteristic length of 1 m is about 1.9 Pa^35^. To the best of our knowledge, this is the first study to observe maintaining macroscale air bubbles at such a highly shear flow condition of *τ*_*w*_ up to 21.6 Pa, which strongly implies a reduction in frictional drag.

## Discussion

In conclusion, we demonstrate a super water-repellent hybrid structure that combines the superhydrophobic, high-aspect-ratio nanowires to effectively grab an air layer in water with the micro-scale denticle structures to reduce the flow resistance by vortices in a highly shear flow. The hybrid structures are fabricated by PDMS molding process of micro-scale denticle structures, hydrothermal growth of high-aspect-ratio nanowires, and coating of a PTFE ultra-thin film. Rotating disk measurements reveal that the hybrid structure performs superior air holding capacity for macroscale air bubbles in a highly shear flow of *τ*_*w*_ up to 21.6 Pa as compared to nanowires alone. These results imply the superior air retention of the proposed structure. In addition, the super water-repellent substrates with the hybrid structures are flexible and attachable onto curved substrates. This feature allows applications to various objects, including ships, pipes and underwater vehicles, that need frictional drag reduction. Our study represents the importance of engineered structures that combine micro- and nano-scale structures as an approach to realize enhanced air retention in industrial applications.

## Methods

### Fabrication of water-repellent hybrid structures

The metallic mold is prepared by using laser processing. Then, the PDMS molding process is performed by pouring the mixture solution of elastomer and curing agent (Sylgard 184, Dow Corning) in a weight ratio of 10:1 onto the mold, and by subsequent curing at 100 °C. Before hydrothermal growth, the surfaces of the flexible PDMS substrate are rendered hydrophilic by using oxygen plasma (a power of 300 W and a frequency of 13.56 MHz, 10–20 min). Hydrothermal growth of cobalt hydroxide carbonate nanowires on the flexible PDMS substrate is carried out by using a mixture of 0.1 M cobalt nitrate hexahydrate (Co(NO_3_)_2_∙6H_2_O, Sigma Aldrich), 0.5 M urea (CO(NH_2_)_2_, Sigma Aldrich), and 0.3 M ammonium fluoride (NH_4_F, Sigma Aldrich) in deionized water^36^. The mixture solution is stirred for 30 min and prepared in an autoclave. The autoclave is heated at 120 °C in an oven for 15 h, while the substrate is placed upside down in the growth solution. Finally, PTFE ultra-thin film is coated on the hybrid structure (*i.e*., cobalt hydroxide carbonate nanowires on the flexible PDMS substrate with micro-scale denticle structure) by dip coating with 1 wt% Teflon AF amorphous fluoroplastic resin (AF2400, Chemours) in fluorinert FC-40 (Sigma Aldrich), followed by heating at 165 °C for 10 min and 245 °C for 5 min. In the oil-repellency experiments, for the better oil-repellency, 0.1 vol% perfluorodecyltrichlorosilane (PFDTS), the self-assembled monolayer (SAM) is additionally modified on the prepared surfaces with PTFE coating. Specifically, after forming hydroxyl group on the surface by using oxygen plasma (a power of 300 W and a frequency of 13.56 MHz, 10–20 min), we immerse the sample in a solution of 0.1 vol% perfluorodecyltrichlorosilane (PFDTS) in n-hexane for 10 min; the sample is then heated at 130 °C for 1 h.

## Electronic supplementary material

Supplementary Information Video S1

Supplementary Information Video S2

Supplementary Information
