# Supplementary material for: Water-repellent Hybrid Nanowire and Micro-scale Denticle Structures on Flexible Substrates of Effective Air Retention
Source: Sci Rep. 2018 Nov 9;8:16631. doi: 10.1038/s41598-018-35075-2 (PMC6226506; doi:10.1038/s41598-018-35075-2)
Supplement: Supplementary file 3 — Supplementary Information [file 41598_2018_35075_MOESM3_ESM.docx]

**Supplementary Information**

Water-repellent Hybrid Nanowire and Micro-scale Denticle Structures on Flexible Substrates of Effective Air Retention

*Sungwon Jo^†,‡^, Seongbin Ahn,^†,‡^, Heungsoo Lee^†^, Chul-Min Jun*g^‖^*, Simon Song^†,§,*^, Dong Rip Kim^†,§,*^*

^†^ School of Mechanical Engineering, Hanyang University, Seoul, 04763, South Korea

^§^ Institute of Nano Science and Technology, Hanyang University, Seoul, 04763, South Korea

^‖^ The 6^th^ R&D Institute, Agency for Defense Development, Changwon, South Korea

^‡^ These authors contributed equally to this work.

* Corresponding author: [*simonsong@hanyang.ac.kr*](mailto:simonsong@hanyang.ac.kr)*,* [*dongrip@hanyang.ac.kr*](mailto:dongrip@hanyang.ac.kr)


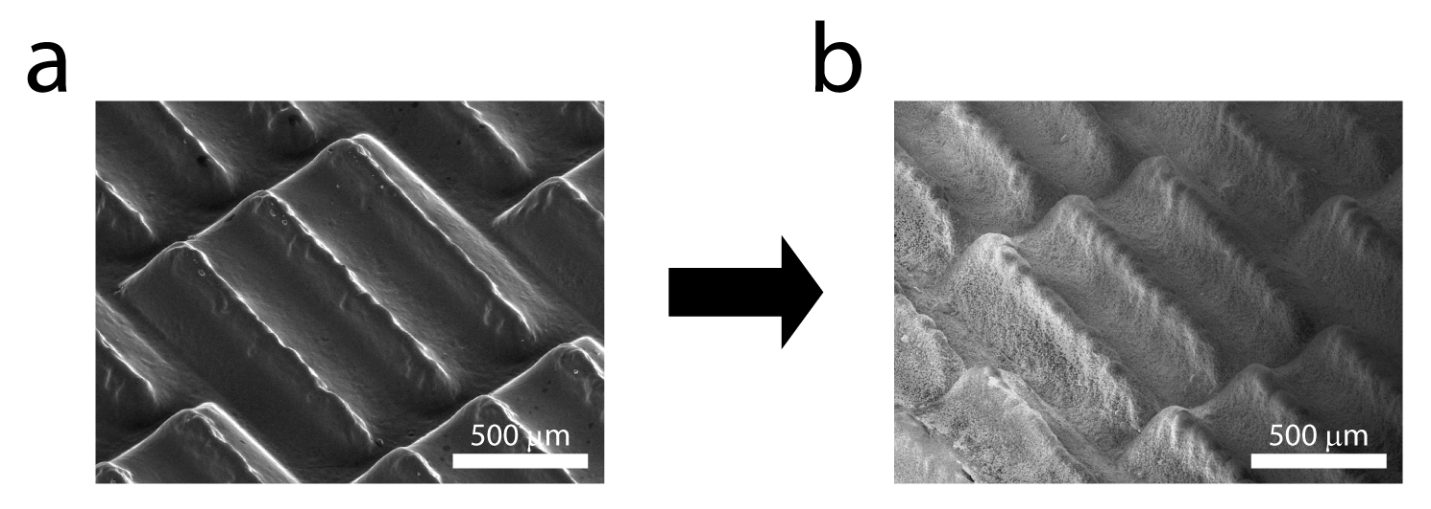


**Figure S1.** Scanning electron microscopy (SEM) images of micro-scale denticle structures (a) before and (b) after hydrothermal synthesis of nanowires and subsequent coating. There is no distinct shape deformation after the synthesis of the nanowire and the coating.


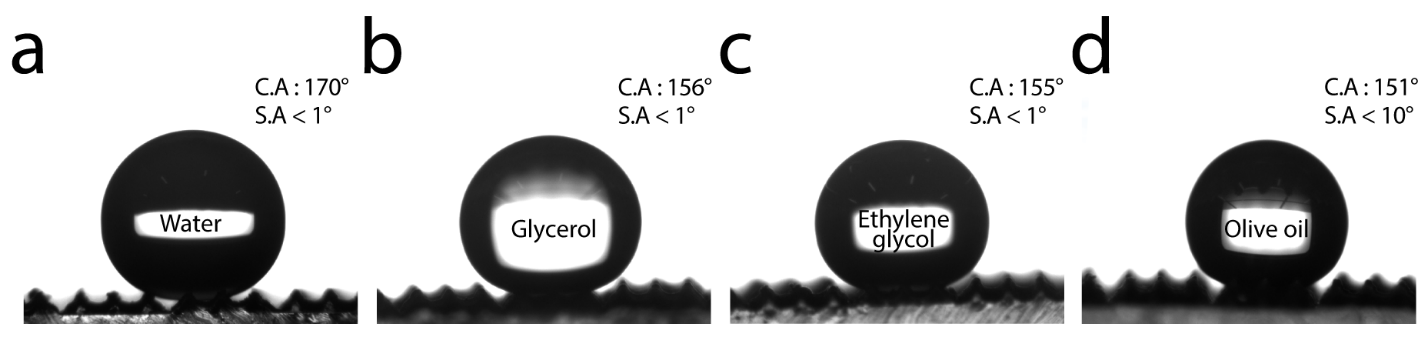


**Figure S2.** Static contact angle measurement of hybrid structures with (a) water, (b) glycerol, (c) ethylene glycol, and (d) olive oil. The oil-repellent structures are fabricated by carrying out the additional modification of the self-assembled monolayer on the hybrid structure.


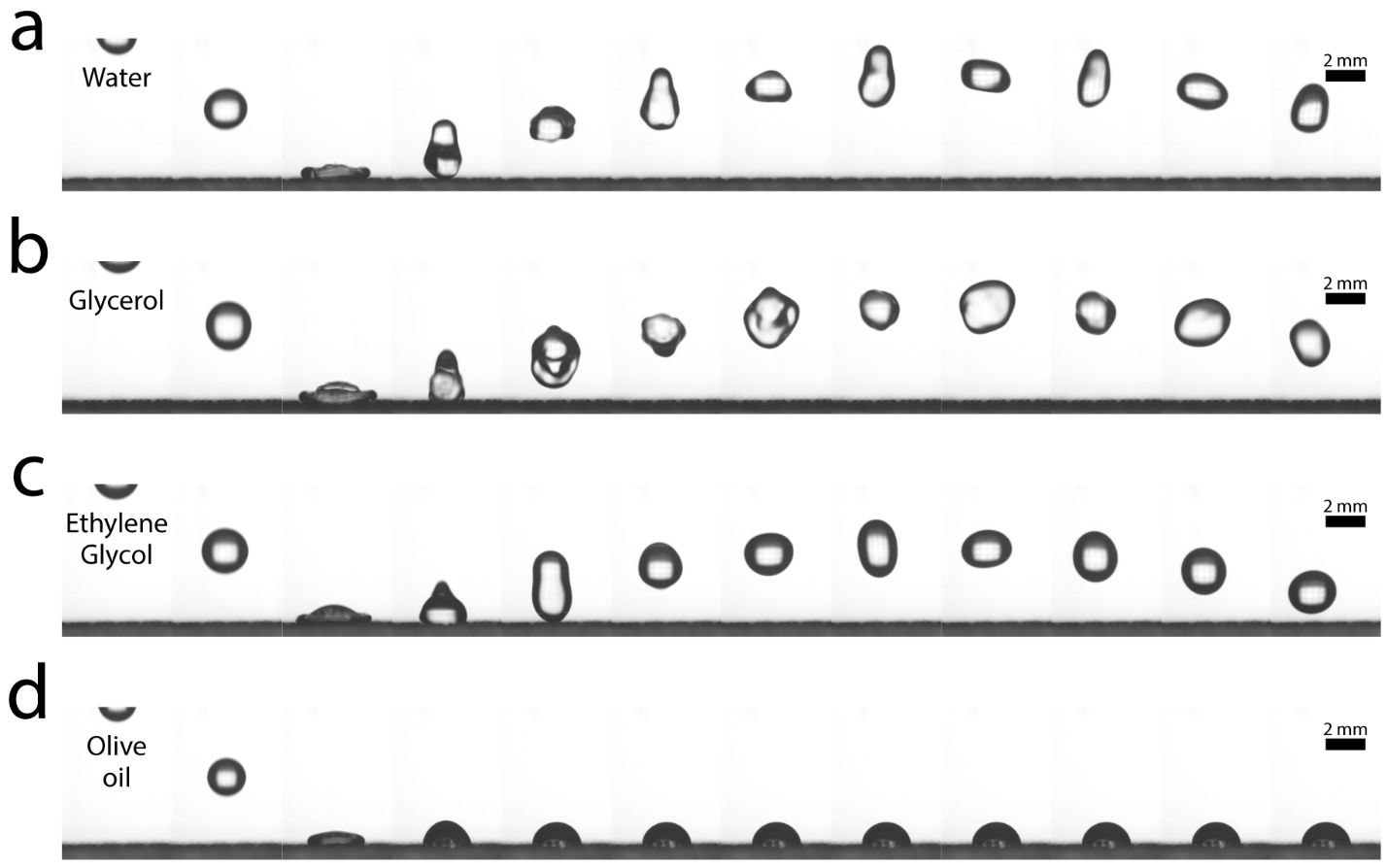


**Figure S3.** Dynamic droplet behaviors of hybrid structures with (a) water, (b) glycerol, (c) ethylene glycol, and (d) olive oil. Behaviors with dynamic droplet of olive oil are measured after the additional modification with perfluorodecyltrichlorosilane (PFDTS) coating. The droplet motion is captured with an interval of 6 ms.


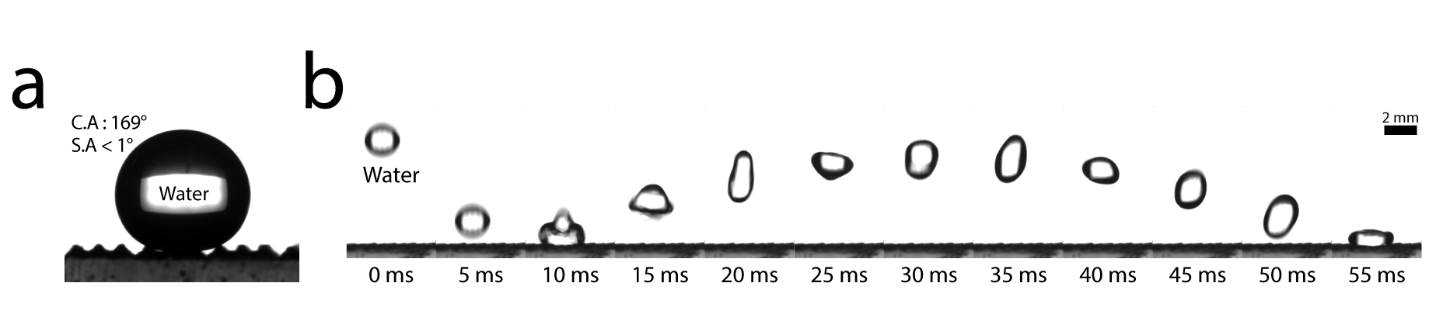


**Figure S4.** Photographs of (a) static contact angle of water and (b) dynamic droplet behavior of the hybrid structures after the salt water immersion test by soaking the sample in the 5 wt% sodium chloride solution for a month.


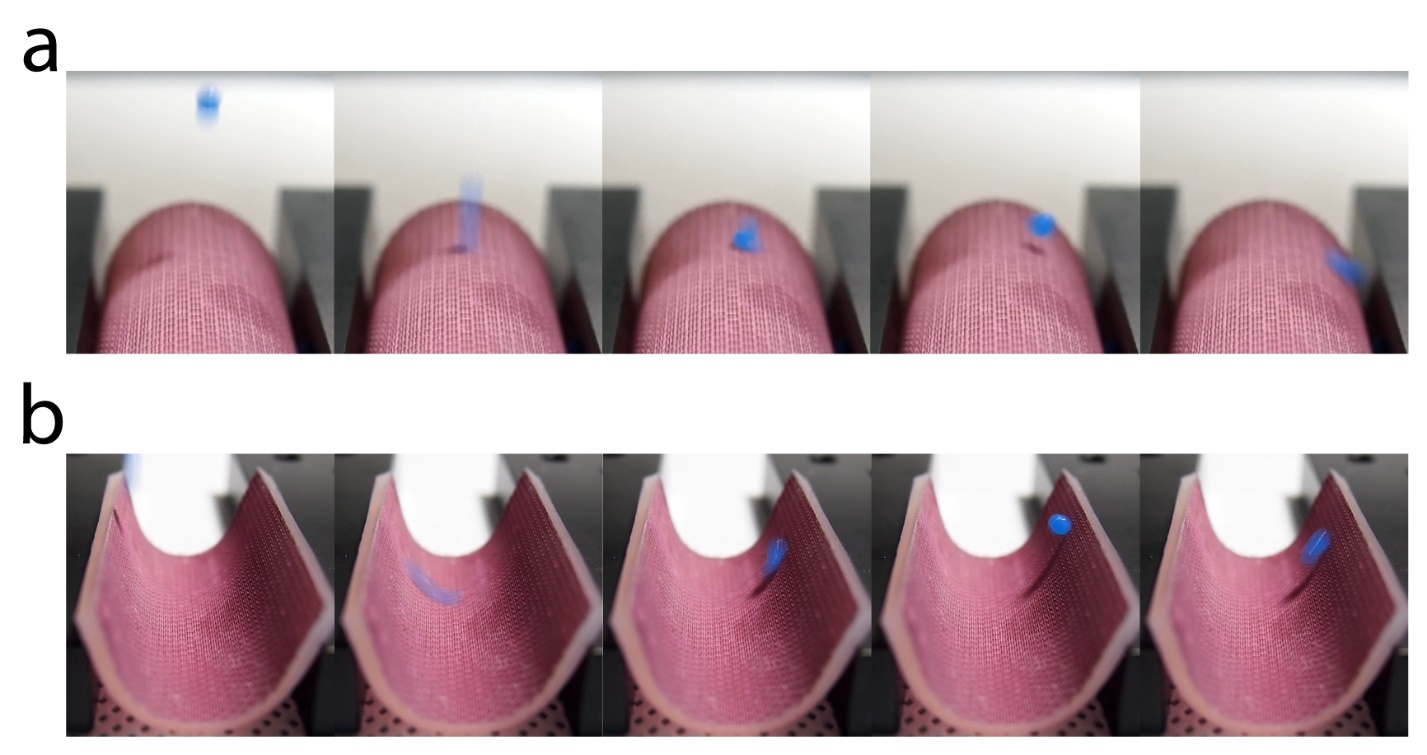


**Figure S5.** Photographs of water droplet behaviors dropping on hybrid structures attached to (a) convex and (b) concave surfaces with the curvature radius of 10 mm.


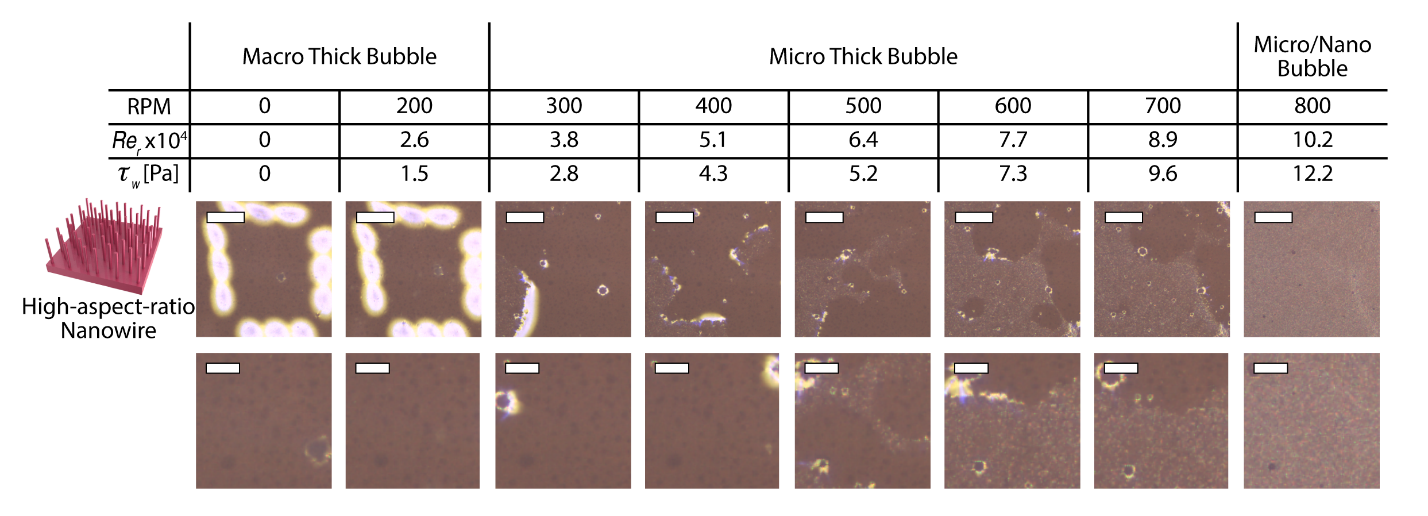


**Figure S6.** Air holding capacity of superhydrophobic, high-aspect-ratio nanowires in dynamic flow conditions of water. Macroscale thick air bubbles can be maintained by the flow conditions of *τ_w_* of 1.5 Pa (*Re_r_* of 2.6×10^4^), and those macroscale thick air bubbles are changed to microscale thick bubbles in the flow conditions of *τ_w_* of 2.8 Pa (*Re_r_* of 3.8×10^4^). In higher flow conditions of *τ_w_* of 12.2 Pa (*Re_r_* of 10.2×10^4^), the nanowires cannot retain the microscale thick bubbles. (scale bars of top images and bottom images are 1 mm and 300 μm, respectively).


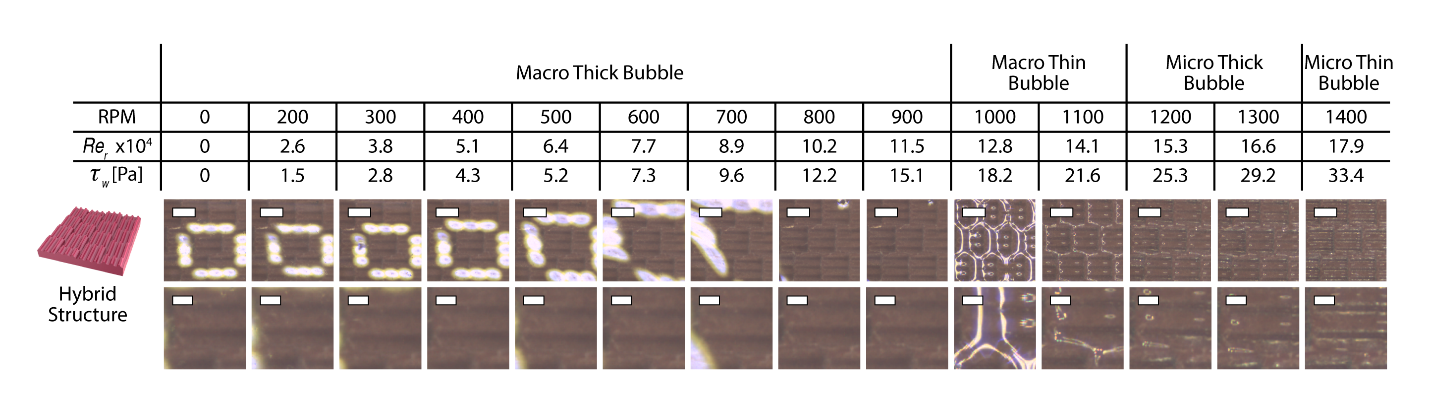


**Figure S7.** Air holding capacity of hybrid structures in dynamic flow conditions of water. The hybrid structures can well maintain the macroscale thick and thin bubbles by the flow conditions of *τ_w_* of 21.6 Pa (*Re_r_* of 14.1×10^4^), and after which the macroscale bubbles are changed to microscale thick air bubbles. The hybrid structures can retain the microscale thick air bubbles by the flow conditions of *τ_w_* of 29.2 Pa (*Re_r_* of 16.6×10^4^). In more dynamic flow conditions, the microscale thick air bubbles are changed to the microscale thin air bubbles on the hybrid structures. (scale bars of top images and bottom images are 1 mm and 300 μm, respectively).

|  | Hybrid structure | Nanowire +AF2400 | Denticle structure +AF2400 | Planar +AF2400 |
| --- | --- | --- | --- | --- |
| Water CA (°) | 170.2 ± 1.8 | 152.2 ± 0.3 | 140.0 ± 0.3 | 118.3 ± 0.4 |
| Glycerol CA (°) | 155.8 ± 1.0 | 149.9 ± 0.9 | 135.7 ± 0.6 | 110.4 ± 0.4 |
| Ethylene glycol CA (°) | 155.3 ± 1.6 | 143.3 ± 0.7 | 125.7 ± 0.5 | 94.8 ± 0.5 |
| γ_s_ (mJ m^-2^) | 0.01 - 0.33 | 0.79 - 0.85 | 3.22 - 3.32 | 12.19 - 13.25 |

**Table S1.** Contact angles and surface energies of surfaces. Surface energy is calculated by using contact angles of each surfaces with water, glycerol and ethylene glycol based on the Li and Neumann method ^1^. To validate the results, we also measure the surface energy of planar with Teflon AF2400 coating. The calculated surface energy of planar with Teflon AF2400 coating agrees well with the previous report ^2^.

1. Li, D. & Neumann, A. W. Contact angles on hydrophobic solid surfaces and their interpretation. J Colloid Interface Sci 148, 190-200, (1992),

2. Tavana, H. & Neumann, A. W. Recent progress in the determination of solid surface tensions from contact angles. Advances in Colloid and Interface Science 132, 1-32, (2007).


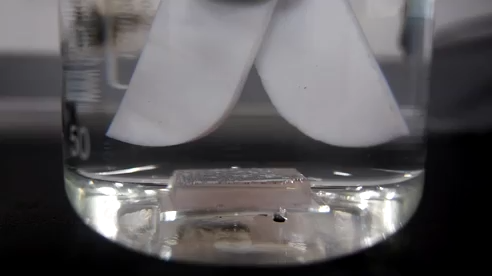


**Video S1.** Air layer holding of hybrid structures in dynamic flow conditions of water.


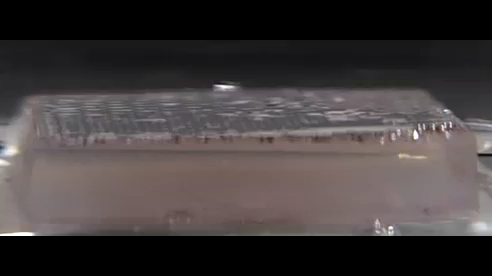


**Video S2.** The high-magnification video of hybrid structures holding the air layer in dynamic flow conditions of water.
